# Full ablation of C9orf72 in mice causes immune system-related pathology and neoplastic events but no motor neuron defects

**DOI:** 10.1007/s00401-016-1581-x

**Published:** 2016-05-20

**Authors:** Emma Sudria-Lopez, Max Koppers, Marina de Wit, Christiaan van der Meer, Henk-Jan Westeneng, Caroline A. C. Zundel, Sameh A. Youssef, Liesbeth Harkema, Alain de Bruin, Jan H. Veldink, Leonard H. van den Berg, R. Jeroen Pasterkamp

**Affiliations:** Department of Translational Neuroscience, Brain Center Rudolf Magnus, University Medical Center Utrecht, Universiteitsweg 100, 3584 CG Utrecht, The Netherlands; Department of Neurology and Neurosurgery, Brain Center Rudolf Magnus, University Medical Center Utrecht, 3584 CX Utrecht, The Netherlands; Department of Pathobiology, Faculty of Veterinary Medicine, Dutch Molecular Pathology Center, Utrecht University, 3584 CL Utrecht, The Netherlands; Division of Molecular Genetics, Department of Pediatrics, University Medical Center Groningen, 9713 AV Groningen, The Netherlands

**Fig. 1:**
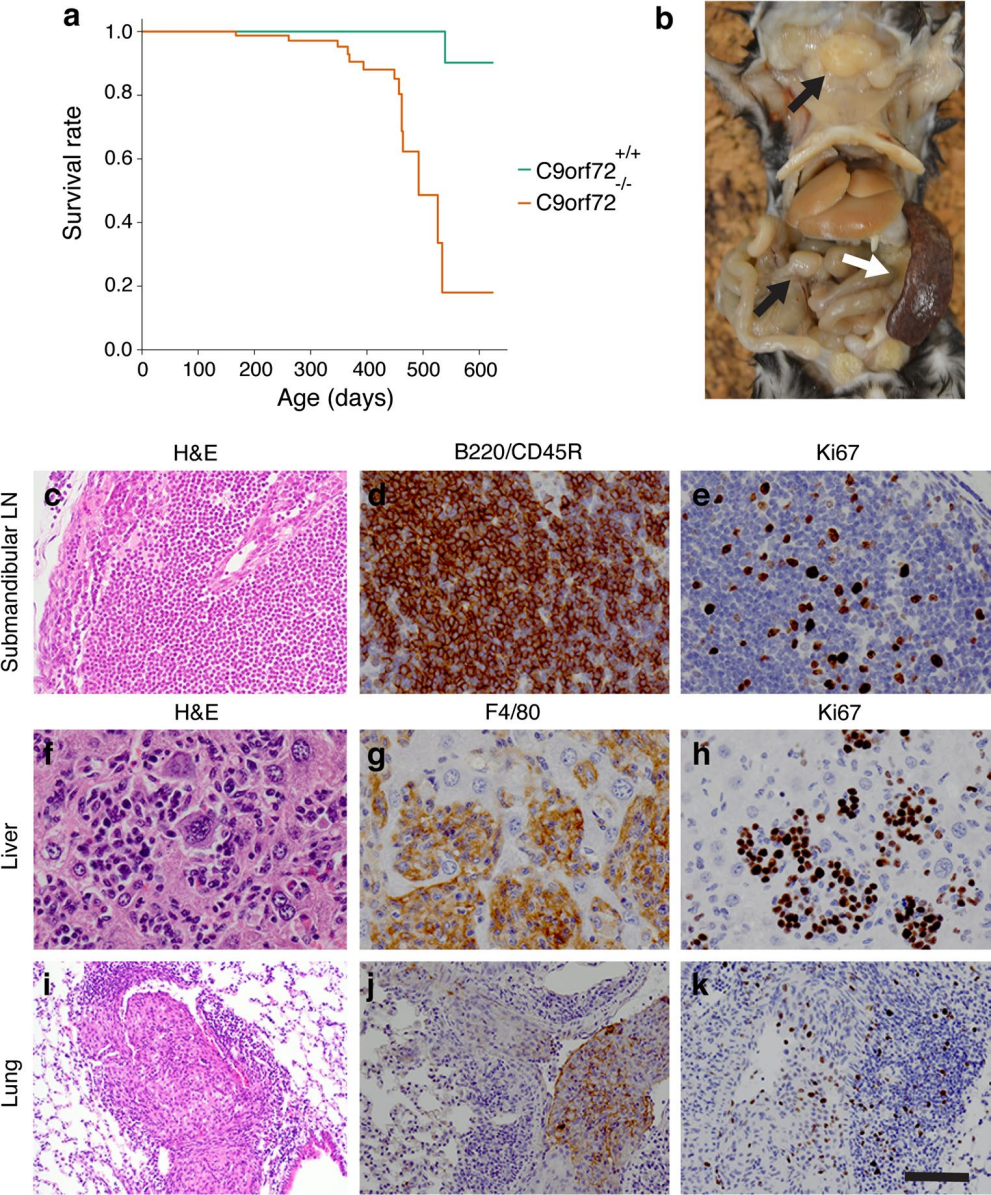
*C9orf72* knockout mice display reduced survival, immune system-related pathology and neoplastic events. **a** Kaplan–Meier curves show survival rates corrected for gender and body weight. *C9orf72* knockout mice show reduced survival as compared to littermate controls (Hazard ratio = 19.0; 95 %, CI: 2.4–150.2, *P* = 0.005). Wild-type controls *n* = 24; *C9orf72* knockout *n* = 29. **b** Gross image showing enlarged lymph nodes (LNs; *black arrows*) and splenomegaly (*white arrow*) in a *C9orf72* knockout mouse (12 months of age). **c**–**e** B-cell lymphoma in the submandibular LNs of *C9orf72* knockout mouse. Nodal tissue is effaced by a monotypic cell population composed of B220/CD45R-positive lymphocytes (B cells). Note the high proliferation rate of the neoplastic lymphocytes as indicated by immunostaining for Ki67 (proliferation marker). **f**–**h** Histiocytic sarcoma in the liver of *C9orf72* knockout mouse. Hepatic sinusoids are filled with atypical histiocytes and multinucleated giant cells that stain positive for the macrophage lineage marker F4/80 and exhibit a high proliferation rate, as evidenced by Ki67 immunostaining. **i**–**k** Histiocytic sarcoma in lung vasculature in *C9orf72* knockout mouse. Pulmonary blood vessels are filled with atypical and multinucleated giant cells that immunostain for F4/80 and Ki67. *H&E* hematoxylin and eosin. *Scale bar* 1.3 cm (**b**), 65 μm (**c**), 40 μm (**d**–**h**), 125 μm (**i**), and 90 μm (**j**, **k**)

Non-coding hexanucleotide (GGGGCC) repeat expansions in *C9ORF72* are the most common genetic cause of amyotrophic lateral sclerosis (ALS) and frontotemporal dementia (FTD; C9ALS/FTD). Decreased C9orf72 protein levels in C9ALS/FTD patients [4] support the idea that C9ORF72 haploinsufficiency may contribute to disease pathogenesis. To test this hypothesis, we previously generated and analyzed neural-specific *C9orf72* knockout mice. Our results showed that neural-specific ablation of C9orf72 (3110043O21Rik) in mice does not cause motor neuron degeneration or changes in motor function [3]. We therefore concluded that loss of C9ORF72 on its own is unlikely to cause ALS and that reducing C9ORF72 levels may comprise a promising strategy to treat C9-ALS patients. This therapeutic potential led us, and others [1, 2], to subsequently analyze knockout mice lacking C9orf72 in all tissues. Importantly, in contrast to our previous report, we find that full ablation of C9orf72 induces reduced survival (Fig. 1a), which is in line with a recent study by Atanasio et al. [1] who report, but do not specify, decreased survival rates. In line with our previous observations [3], full C9orf72 ablation results in a 5.9 % decrease in body weight (*P* = 0.0056), without affecting motor function (accelerating rotarod and grip strength test) or inducing pathological hallmarks of ALS (see also [1, 2]), such as motor neuron degeneration, gliosis, enhanced ubiquitination and TDP-43 mislocalization. However, post-mortem analysis of full *C9orf72* knockout mice (*n* = 5; 11–15 months of age) revealed enlarged lymph nodes (LNs) (*n* = 4 mice) and splenomegaly (*n* = 5) (Fig. 1b). Detailed histological evaluation detected massive infiltration of histiocytes/macrophages and lymphocytes in multiple organs, including LNs, spleen, bone marrow, liver, kidney and lung (Fig. 1c–k). In addition to these immunological phenotypes, which are in part also reported by Atanasio et al. [1] and O’Rourke et al. [2], we detect evidence of neoplastic events. LNs of several animals (*n* = 4) contained infiltrates of B220/CD45R-positive B-lymphocytes that disrupted tissue architecture and were accompanied by increased expression of the proliferation marker Ki67, suggesting the development of B-cell lymphomas (Fig. 1c–e). Furthermore, disrupted tissue architecture and homogeneous populations of F4/80-positive macrophages expressing Ki67 were present in LNs (*n* = 3), spleen (*n* = 3), liver (*n* = 1) and lung (*n* = 1). Moreover, infiltrating cells in the liver and lung accumulated in intravascular spaces (Fig. 1f–k), suggesting the occurrence of metastatic histiocytic sarcomas. These results indicate that the defects in immune cell function recently reported in *C9orf72* knockout mice (e.g. changes in endosome/lysosomal trafficking, cytokine production) [1, 2] may ultimately lead to neoplastic events in multiple organs. These findings have important implications as they indicate that strategies aimed at lowering systemic C9ORF72 levels in C9ALS/FTD patients may have negative side effects and that emphasis should be on therapeutic approaches that selectively target the hexanucleotide repeat expansions or their downstream pathogenic effects.
